# Dicussion upon the Desirability of Extraction of the Sixth-Year Molar

**Published:** 1892-12

**Authors:** 


ARTICLE IX.

### DISCUSSION UPON THE DESIRABILITY OF EXTRACTION OF THE SIXTH-YEAR MOLAR.

The chairman, in introducing a discussion on "The Desirability of Extraction of the Six-year-old Molar," said the subject was of the greatest interest and importance in the treatment of youthful mouths. The subject might be considered generally as the treatment of irregularities and overcrowding of the human teeth. Their special point for discussion, however, was the tendency of overcrowding to prevent the destructive effects of caries, whether it might be justifiable as a conservative measure in cases, where they saw indications of danger to the youthful denture from this cause, to remove an upper and a lower tooth from each side of the mouth with a view to the giving of space to the anterior teeth, permitting them to settle, and as nearly as possible, isolate themselves, thereby preventing the inception of carious disease in such teeth, as had not already been attacked; and if such a measure was justifiable, which teeth should be selected for extraction so as to give the necessary relief, and when would be the best time for extracting to secure the best results for the future of the mouth.

Dr. I. B. Davenport (Paris) said it was deplorable to realize how little was known of the true function of the organs of mastication. Men constantly attempted the treatment of cases of irregularity with their ideas of where the imperfection lay, and of what would constitute perfection quite reversed, and it was not surprising that disappointment resulted. His researches showed him that it was at first necessary to demonstrate so obvious a fact as that lateral contact of the teeth was an essential factor in a normal arch, and that good arches could not be disturbed without injury. This he was obliged to do because thousands of dentists the world over were mutilating the teeth both by extracting and cutting between them, on the theory that "contact is dangerous," and who did not realize the consequences of broken arches, and even to-day that fact was not enough appreciated. Good dental arches possessed such an arrangement as to be mutually self-supporting, thus tending to their own preservation and highest functional power. If a portion of the dental arches were removed, they fell together, and the new arches thus formed were apt to be unstable for want of proper support, and often pushed out into irregular shapes at the points of feeble resistance. When the arches fell together after extraction the roots of the teeth moved less than the crowns, and so the relations of the grinding surfaces were disturbed, and much of the interlocking support of the masticating surface was lost, and a large space was left, which required much diminution in the size of the arch to obliterate. When the jaws were at rest there ought to be broad and firm contact between the two arches all around, and during any masticating movement there should be extensive progressive contact, as the articulation moved from one place to another. He had been criticised for insisting upon the application of knowledge of perfect or good arches in considering the results of treatment of imperfect arches, and it had been claimed that a correct judgment of a case could be reached only by comparison of the jaws before and after extraction. No source of knowledge could be despised, and he was as much in favor of employing that method of research as anybody could be. Much might be learned by comparing results with previous conditions, but the value of an opinion by such comparison as to whether improvement or injury had been done, as well as the ability to detect them, must inevitably depend upon the knowledge they possessed of what constituted a perfect denture. There were perfect elements of error in the comparison of results before and after extraction. What might appear to be a better result at one period might be bad later, after the unbalanced forces had had a longer time to exert their influences. Take the slowly developing child that seemed to be "all teeth," a comparison of photographs years after extraction with one showing the previous condition of the child would be valueless as indicative of the degree of improvement of the features. Such were the cases with which some dentists delighted to dabble. The parents thought "the girl horrid," and "something must be done" they said. The too-supine dentist extracted the teeth, and after a time compared the two conditions as shown by models, and parents, dentist and all were happy. To contrast the actual results obtained with models of the past conditions without taking cognisance of the possible and not improbably changed condition produced by time alone, was, in his opinion, unscientific at least.

Mr. G. C. Campion (Manchester) said they would find in the new books which had been prepared for the use of the School Investigation Committee, that carious teeth were classed into two categories. There were carious saveable teeth, and carious unsaveable teeth, and among the latter were a large number of six-year old molars. When it was proposed to illustrate the results of the extraction of the six-year-old molar it seemed to him and others that it would be a useful thing to have some models showing the results of extraction of those teeth at different times, so that they might find out under what conditions of the development of the denture it was best to remove those carious unsaveable molars in order to produce the best possible results in after years. In photographs of models which have been taken in cases where the six-year-old molars had been removed, what he thought they would notice was that where the lower molar alone had been removed and the upper left the result upon articulating was very bad, and that where the six-year-old molars above and below had been taken out the result was very much better. In future cases, dentists ought to make a point of taking models before molars were removed as well as afterwards. By such a course they might gather a large amount of evidence upon the point which would be of great practical utility.

Mr. G. Cunningham (Cambridge) said that in the university town where he lived he had ample opportunity of seeing how great was the neglect of the teeth among the young men of the country. He quite agreed with previous speakers as to the necessity of models being taken to establish the desirableness or otherwise of removing the six-year-old molars, in cases of irregularity, and he produced a number of highly ingenious models which he had himself made.

Mr. C. S. Tomes (London) said the dental arch theory was untenable in the present case, because the key stone of an arch was not in its side. It was the side of the denture where the force of mastication came and they were talking outside the mark when they talked about the spring of the arch. Formerly he used to take out the six-year-old molars in case of irregularity, but now he would rather take out a sound bicuspid than a somewhat damaged six-year-old molar.

Mr. W. B. Pearsall (Dublin) said he looked upon the man who extracted six-year-old molars, if they were sound, as a criminal. He held that a bicuspid could much better be sacrificed.

Mr. Mundell said he had recently examined 160 boys in an important school and had found the most of them would have been benefitted by the extraction of the six-year-old molar. Much depended upon the constitution of the teeth. If the teeth were weak, he held that they should extract the molars so as to allow the bicuspids to be useful in after life.

Mr. F. Rose and Mr. Storer Bennett also spoke.

The Chairman, in bringing the discussion to a close, said the great end every dentist should aim at was the preservation of the greatest number of teeth in the soundest possible condition.—*Dental Record.*

